# Letter to the Editor: Corneoscleral Melt 50 Years after Excision of Pterygium

**DOI:** 10.2174/1874364101711010047

**Published:** 2017-03-31

**Authors:** Aki Kondo, Tatsuya Mimura, Mari Goto, Yuko Kamei, Saito Yusuke, Hiroko Okuma, Masao Matsubara

**Affiliations:** Department of Ophthalmology, Tokyo Women's Medical University Medical Center East, Tokyo, Japan

**Keywords:** Corneal perforation, Mitomycin C, Pterygium, Cataract surgery, Penetrating keratoplasty, Scleral melt

## Abstract

**Purpose:**

To report a case of corneoscleral melt that occurred 50 years after resection of pterygium with postoperative administration of mitomycin C (MMC).

**Results:**

A 93-year-old woman developed acute corneal perforation and scleral melt in her left eye at 50 years after pterygium surgery with postoperative topical MMC. She underwent limbal transplantation. The anterior chamber reformed postoperatively and her intraocular pressure was normal. At 12 months after transplantation, best-corrected visual acuity was 20/500 and the graft-host junction was well apposed.

**Conclusion:**

This case shows that corneoscleral melt can occur even 50 years after resection of pterygium combined with postoperative topical MMC.

## INTRODUCTION

Pterygium is a chronic condition characterized by fibrovascular overgrowth of conjunctival tissue onto the cornea. The only available treatment is surgical resection. Mitomycin C (MMC) is a useful adjunct to pterygium surgery, being employed to prevent recurrence of pterygium after excision [1-4]. However, recent reports have suggested that adverse reactions such as scleral melting and corneal perforation can be associated with the use of MMC [5-8]. We describe an extremely rare case of corneoscleral melt that occurred 50 years after pterygium surgery with postoperative topical MMC.

## CASE REPORT

**Fig. (1):**
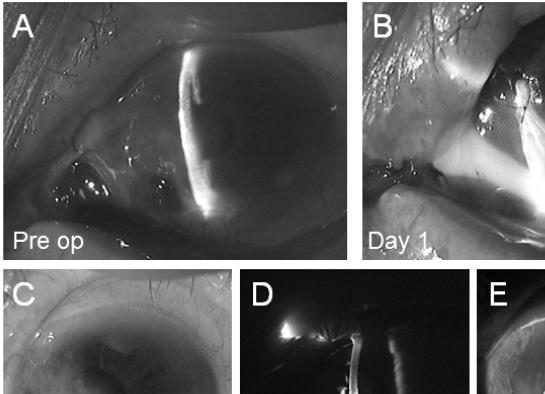
(A) Slit-lamp photograph: perforation of the nasal cornea sealed with iris tissue and collapse of the anterior chamber in a 93-year-old woman. (B) Postoperative appearance: a deep anterior chamber has reformed at one day after penetrating keratoplasty. (C-E) At 1 year postoperatively, the cornea at the surgical site shows complete healing and re-epithelialization, along with mild thinning and stromal opacity at the site of the previous corneoscleral melt.

A 93-year-old woman was referred to our outpatient clinic for management of acute corneal perforation in the left eye. She had undergone surgery for pterygium of the left eye at the age of 44 years and was treated with topical MMC postoperatively, although she could not recall the MMC concentration used or the duration of administration. Cataract surgery had also been performed on the left eye at the age of 84 years. She had no history of visual problems or symptoms, and there was also no history of diabetes or collagen diseases including rheumatoid arthritis. One month earlier, she had complained of pain in the left eye and a limbal corneal ulcer had been diagnosed by her primary care physician. She was treated with topical medications, but the ulcer became progressively worse and corneal perforation occurred. At her first visit, best-corrected visual acuity was 20/60 in the right eye and counting fingers in the left eye. The intraocular pressure (IOP) was 19 mmHg on the right and 6 mmHg on the left. Slit lamp examination of the left eye revealed a corneal ulcer near the inferior nasal limbus and collapse of the anterior chamber Fig. (**1A**). In addition, the uvea near the limbus showed dome-shaped prolapse under the conjunctiva. Focal stromal scarring from previous cataract surgery was seen in the superior temporal limbus. On the next day, urgent penetrating keratoplasty was performed using a donor cornea together with vitrectomy. The graft was attached to the nasal cornea and sclera of the patient with interrupted 10-0 monofilament nylon sutures. A transfixing suture could not be placed at the site of perforation because of corneal melting and thinning. The conjunctiva had covered the exposed uveal tissue in the region of the inferior nasal scleral defect, so additional scleral patch grafting was not performed. The anterior chamber reformed on the first day after surgery Fig. (**1B**). Her postoperative course was uneventful and visual acuity of the affected eye was 20/500 at 12 months after surgery (Figs. **1C**-**1E**).

## DISCUSSION

In this patient, we suspect that corneoscleral perforation with iris prolapse may have resulted from the use of topical MMC after pterygium surgery. Unfortunately, the details of her MMC treatment were unknown because it was performed 50 years earlier. In Japan, MMC has been used to treat pterygium for more than 50 years since the first attempt at employing topical MMC to prevent recurrence of pterygium after surgery was reported in 1963 by Kunitomo and Mori [9]. At that time, the effects of MMC and the optimum concentration and application time were unknown. Thus, use of topical MMC might not have been appropriate in some patients from the 1960s to the early 1980s.

MMC inhibits conjunctival fibrovascular proliferation and migration of Tenon's fibroblasts after surgery. Thus, MMC treatment can decrease postoperative recurrence of pterygium and improve surgical outcome [10]. In the late 1980s and early 1990s, an increase in the popularity of MMC was reported in the United States by Singh and associates [11]. However, use of topical MMC to prevent recurrence of pterygium after excision was reported to be associated with corneoscleral melting [5, 6]. Rubinfeld and associates reported a series of 10 patients who experienced serious vision-threatening complications, including secondary glaucoma (4 patients), corneal edema (3 patients), corneal perforation (1 patient), corectopia (2 patients), iritis (8 patients), sudden-onset mature cataract (2 patients), and scleral calcification (1 patient), following the use of topical MMC after pterygium surgery [5]. They recommended avoiding MMC or using a very low concentration for patients with ocular complications such as dry eye and poor wound healing [5]. Despite this report, intraoperative application of MMC to the scleral bed has been widely used in pterygium surgery [12-15].

The reported interval between the occurrence of corneoscleral melt and resection of pterygium with topical application of MMC varies between eight days and 16 years [4, 7, 8]. Our patient developed acute corneal perforation and scleral melt at 50 years after pterygium surgery. An age-related increase in the fragility of the extracellular matrix may also have been associated with corneoscleral melt in this case.

## CONCLUSION

In conclusion, we experienced a rare case of corneoscleral perforation at 50 years after surgical treatment of pterygium. It is necessary to take into consideration the possibility that perforation may occur a very long time after pterygium surgery.

## PROPRIETARY INTEREST

The authors do not have any proprietary interest in this manuscript.
